# Text information hiding and recovery via wavelet digital watermarking method

**DOI:** 10.1038/s41598-023-36759-0

**Published:** 2023-06-12

**Authors:** Zhou Xiaohui

**Affiliations:** Shanghai University of Finance and Economics-Zhejiang College, Jinhua, 321013 Zhejiang China

**Keywords:** Electrical and electronic engineering, Mathematics and computing, Applied mathematics, Information technology

## Abstract

According to the wavelet digital watermarking method, wavelet text hiding algorithm is presented for hiding some text information in a signal with white noises and the corresponding recovery algorithm is also presented for obtaining text information from a synthesized signal. Firstly, wavelet text hiding algorithm is introduced and an example is given for demonstrating how to hide text information in a signal *s* with a white noise *ε*, where *s* = *f*(*x*) + *ε* and *f*(*x*) is a function such as sin *x*, cos *x* and so on. A synthesized signal $\tilde{s}$ can be obtained by wavelet text hiding algorithm. Then, the corresponding text recovery approach is also introduced and the text information is recovered from the synthesized signal $\tilde{s}$ by an example. Some figures of the example are shown that the wavelet text hiding algorithm and its recovery are feasible. Moreover, the roles of wavelet function, noise, embedding mode and embedding position are analyzed in the text information hiding and recovering, and it implicates its security. 1000 groups of English texts with different lengths are chosen for illustrating computational complexity and running time of the algorithms. The social application of this approach is explained by a system architecture figure. Finally, some future directions are discussed for our follow-up study.

## Introduction

As it is known to all that the study of cryptography has been a hot spot in the field of information security, and widely used in the military, national security, business, electronic industry and many other fields. It mainly includes encryption and decryption. It is of great significance to protect the real information and data from theft by invaders or hackers. According to the communication theory of secrecy systems presented by Shannon^1^, cryptography technology had been pushed to the scientific track during thousands of years, and a real cryptography had been established. Based on new directions in cryptography discussed by Diffie and Hellman, it was started to study the modern cryptographic algorithm. Two years later, Rivest^2^ proposed the RSA public key cryptosystem, which is the first safe and practical public key cryptography algorithm. RSA is based on number theory. Mathematical methods play an important role in modern cryptographic algorithms, such as SM4 packet password algorithm, SM2 public key password algorithm, SM3 password miscellaneous algorithm and so on^3^. With the development of modern cryptography, scholars have proposed various interdisciplinary encryption methods to protect information security, web and mobile communication security, image watermarking security, smart city applications and other aspects in recent years, such as machine learning approaches, data leakage prevention method, anomaly detection method and so on. Song and Yang have discussed a general semi-supervised scene classification method for remote sensing images based on clustering and transfer learning^4^. Gaurav et al. have investigated a comprehensive survey on machine learning approaches for malware detection in IoT-based enterprise information system^5^. Almomani et al. have studied how to Phish website detection with semantic features by machine learning classifiers^6^. In addition to machine learning methods, scholars have also explored other approaches^7–12^. For example, Yu et al. have discussed a data leakage prevention method by the reduction of confidential and context terms for smart mobile devices^7^. Wang et al. have studied an IBN-based location privacy preserving scheme for IoCV^8^. Yu et al. have investigated an edge computing based anomaly detection method in IoT industrial sustainability^9^.

Since the wavelet analysis was born in1980s, wavelet method has been applied widely in many fields^13–15^, such as the signal analysis and processing, the image compression, pattern recognition, detecting the mutation signal, the military electronic countermeasure, economy and finance^16–18^, information security^19,20^ and so on. An important feature of wavelet transform is that it can process non-stationary data, localize in time domain, and perform multiscale analysis for a signal. Faramarz Fekri, Farshid Delgosha have discussed the finite-field wavelets with applications in cryptography and coding systematically, such as wavelet block cipher, wavelet self-synchronizing cipher and so on^20^. Recently, wavelets with applications in cryptography have been developed constantly. The multi-resolution analysis of wavelet transform is also widely used in information security, such as encrypting an audio file by ignteger wavelet transform and hand geometry^21^, hiding reversible data in encrypted images by IWT and chaotic system^22^, the visible digital watermark by integer wavelet transform^23^ and so on. The safe transmission of text information is of great significance in business activities, communication of text information and so on. In these activities, we hope that the most important information can be delivered by a beautiful melody without being found by others. In this paper, an approach is presented to hide and recover the text information in a signal with a noise by wavelet transform.

This paper is organized as follows: in Section "Preliminary", Some preliminaries are illustrated for our discussion, including orthogonal multi-resolution analysis, digital watermarking based on wavelet transform; in Section "An algorithm for hiding a text information", an algorithm for hiding some text information is given by the wavelet method, and an example is shown for demonstrating how to hide text information in a signal. In Section "Recovery algorithm for text information", an approach is presented to recover the text information from the synthetic signal in Section "An algorithm for hiding a text information". Moreover, the roles of wavelet function, noise, computational complexity and running time, embedding mode and position are analyzed in the text information hiding and recovering. Some figures are shown for our discussion.

## Preliminary

### Multi-resolution analysis^13,17^

If a closed subspace sequence $\left\{ {V_{j} } \right\}$ in space $L^{2} (R)$ satisfies the following properties:

1. $$V_{j} \subset V_{j + 1}$$, $$\forall j \in Z$$
2. $$\bigcap\nolimits_{j \in Z} {V_{j} } = \{ 0\}$$, $$\overline{{\bigcup\nolimits_{j \in Z} {V_{j} } }} = L^{2} (R)$$
3. $$f(x) \in V_{j} \Leftrightarrow f(2x) \in V_{j + 1}$$
4. There exists a function $\phi (x)$, such that the set $\{ \phi (x - k),k \in Z\}$ is an orthogonal basis of $V_{0}$. Then, an orthogonal multi- resolution analysis is generated by the closed subspace sequence $\left\{ {V_{j} } \right\}$, where

$$V_{j} = clos_{{L^{2} (R)}} < \phi_{j,k} = 2^{j/2} \phi (2^{j} x - k):k \in Z >$$, $$\phi_{j,k} = 2^{j/2} \phi (2^{j} x - k)$$.

Note 1: From property (1) to property (4), they are consistent monotony, asymptotic completeness, scaling regularity. the existence of orthogonal bases, respectively. The information of the signal can be encoded at the resolution level *j* in each subspace *V*_*j*_. Vector space generated by scaling functions with high resolution level contains that by lower resolution level (More details can be seen in^13,17^).

For each integer $j \in Z$, there exists an orthogonal complementary space $W_{j}$ of $V_{j}$ in the space $V_{j + 1}$, that is, $V_{j + 1} = V_{j} \oplus W_{j}$. Thus,$\oplus_{j \in Z} W_{j} = L^{2} (R)$. If the set $\{ \psi (x - k),k \in Z\}$, generated by a function $\psi (x) \in L^{2} (R)$, is an orthogonal basis of $W_{0}$, where.

$$W_{j} = clos_{{L^{2} (R)}} < \psi_{j,k} = 2^{j/2} \psi (2^{j} x - k):k \in Z > ,$$ then the function $$\phi (x) \in$$$$L^{2} (R)$$ is the scaling function, and $$\psi (x) \in L^{2} (R)$$ is the wavelet function corresponding to $$\phi (x)$$. Thus, the scaling function $$\phi (x)$$ and wavelet $$\psi (x)$$ satisfy the following two-scale equation:

$$\begin{gathered} \phi (x) = \sqrt 2 \sum\limits_{k \in Z} {p_{k} \phi (2x - k)} , \hfill \\ \psi (x) = \sqrt 2 \sum\limits_{k \in Z} {q_{k} \phi (2x - k)} , \hfill \\ \end{gathered}$$

where the sequences $\left\{ {p_{k} } \right\}$ and $\left\{ {q_{k} } \right\}$ are called the low-pass filter and high-pass filter of $\phi (x)$ and $\psi (x)$, respectively.

The decomposition and reconstruction algorithm play an important role in the application of wavelet analysis. For an signal $$f(x) \in$$$$V_{j + 1} \subset L^{2} (R)$$, decomposition algorithm is given as follows:

1 $$\left\{ \begin{gathered} c_{j,k}^{{}} = \sum\limits_{n \in Z} {p_{n - 2k}^{{}} c_{j + 1,n}^{{}} } , \hfill \\ d_{j,k}^{{}} = \sum\limits_{n \in Z} {q_{n - 2k}^{{}} c_{j + 1,n}^{{}} } . \hfill \\ \end{gathered} \right.$$

And reconstruction formula is

2 $$c_{j + 1,n}^{{}} = \sum\limits_{k \in Z} {p_{n - 2k}^{{}} c_{j,k}^{{}} + q_{n - 2k}^{{}} d_{j,k}^{{}} }$$

where

$$\begin{gathered} c_{j,k}^{{}} = < f(x),\phi_{j,k} (x) > ,d_{j,k}^{{}} = < f(x),\psi_{j,k} (x) > , \hfill \\ \phi_{j,k} (x) = 2^{\frac{j}{2}} \phi (2^{j} x - k),\psi_{j,k} (x) = 2^{\frac{j}{2}} \psi (2^{j} x - k). \hfill \\ \end{gathered}$$ The coefficients $c_{j,k}^{{}}$ captures the low-frequency information of the signal $f(x)$, and the coefficients $d_{j,k}^{{}}$ captures the high-frequency information of the signal $f(x)$.

### Digital watermarking based on wavelet transform

Digital watermark has become a hot spot in the security research of multimedia information, and it is also an important branch in the field of information hiding technology research. Digital watermarking technology is mainly used in ticket anti-counterfeiting, copyright protection, tampering tips and hidden signs. The ticket anti-counterfeiting watermark is a kind of special watermark, which is mainly used for the anti-counterfeiting of printed bills, electronic bills and various certificates. The copyright mark watermark is one of the most studied digital watermarks at present. Digital works are both goods and knowledge works. This duality determines that copyright logo watermarking mainly emphasizes invisibility and robustness, but requires relatively little data. Tamper hint watermarking is a fragile watermark, which aims to identify the integrity and authenticity of the original document signal. The purpose of hidden identification watermarking is to hide the important labels of confidential data and limit the use of confidential data by illegal users. Digital watermarking based on the transform domain is the mainstream of the current digital watermark technology research. However, the wavelet transform is widely used in digital watermarking, such as digital audio watermarking^24^, digital ECG signal watermarking^25^, color image watermarking^26,27^ and so on. A digital watermarking algorithm based on a discrete wavelet transform is briefly introduced in this section.

**Figure 1:**
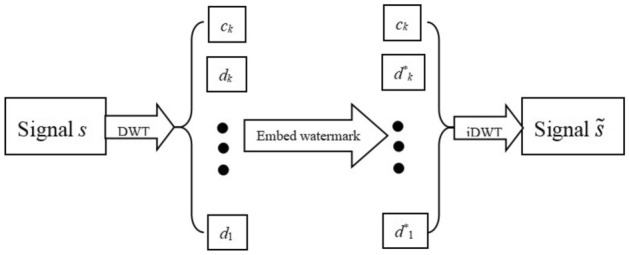
Schematic illustration of the watermark embedding.

For a one-dimension signal $s$, it can be decomposed to a low-frequency component $c_{1}$ and a high-frequency component $d_{1}$ by first discrete wavelet transform. Then the low-frequency component can also be decomposed to a low-frequency component $c_{2}$ and a high-frequency component $d_{2}$ by second discrete wavelet transform. Analogously, a low-frequency component $c_{k}$ and *k* high-frequency components $d_{1} ,d_{2} , \ldots ,d_{k}$ can obtained after *k*-th discrete wavelet transform (see the left of Fig. 1). The low-frequency component $c_{k}$ is an approximation for the original signal. High-frequency components $d_{1} ,d_{2} , \ldots ,d_{k}$ are details of the different frequency bands. Choose an initial position, and a watermarking signal can be embedded in the low-frequency component $c_{k}$ and high-frequency components $d_{1} ,d_{2} , \ldots ,d_{k}$. By inverse discrete wavelet transform, a signal $\tilde{s}$ with a watermarking can be obtained (see the right of Fig. 1). If robustness or encryption should be considered, some approach can be adopted to solve these problems such as different weighting for low-frequency and high-frequency coefficients, embedding an encrypted watermarking and so on. Many Scholars have done much research work.

## An algorithm for hiding a text information

According to the above instruction, the ideology of public key mechanism and wavelet digital watermarking method play an important role in ensuring information security. In this section, we introduce the text information hiding algorithm for information transmission security by the wavelet transform and ideology of public key mechanism. Our main idea is to blend white noise with text information, then it is embedded in a signal as a watermarking. The following algorithm is given firstly:

### Wavelet text hiding algorithm (WTHA)

The first step is to establish an data set for an English text and encode it;

$$\{ [\begin{array}{*{20}c} {m_{1} } & {m_{2} } & \ldots & {m_{l} } \\ \end{array} ]:m_{i} \in Z^{ + } ,i = 1,2, \ldots l\}$$ where *l* denotes the length of the English text;

The second step is to encode the transmitted text information to generate an array;

The third step is to select a signal containing noise.

$$s = f(x) + \varepsilon, \,\, {\rm where}\,\,\varepsilon \sim N(0,\sigma^{2} )$$

and decompose it to *k* levels by the discrete wavelet transform(DWT). we obtain the low frequency coefficient $c_{k}$ and several high frequency coefficients $d_{1} ,d_{2} , \ldots ,d_{k}$;

The fourth step is to take an linear transform on the text information code to make it conform to a certain high frequency coefficient feature, and select the appropriate position to add the transformed coding information to the high frequency coefficient;

The fifth step is to reconstruct the new high frequency coefficients and the low frequency coefficient by the wavelet reconstruction formula to generate the signal with text information code.

Note 2: The linear transform in Step 4 may be invertible for a simple way. To improve higher security, some generalized reversible linear transformations can also be considered. Of course, Adding text code segments to different high frequency coefficients is also a feasible way to improve higher security. These approaches will be discussed in our follow-up study. In this paper, text code is added to only one high frequency coefficient. It is relative simpler and faster.

**Figure 2:**
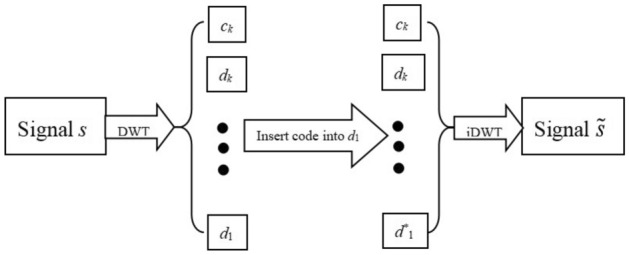
Schematic illustration of Hiding Algorithm for text information.

The above step hides the text information in a signal with white noise. This approach is called the wavelet text hiding algorithm. The diagram of this approach is shown in Fig. 2, the text information is embedded in high frequency coefficients $d_{1}$.

The following example is given for illustrating the wavelet text hiding algorithm.

#### Example 1

Firstly, according to the Algorithm 1, establish an English letter database.

‘ABCDEFGHIJKLMNOPQRSTUVWXYZabcdefghijklmnopqrstuvwxyz… ’.

Choose a text “Text Hiding and Recovery”.

Secondly, the code data of “Text Hiding and Recovery” is an array as follows:

‘[20,31,50,46,53,8,35,30,35,40,33,53,27,40, 30,53,18,31,29,41,48,31,44,51]’.

Thirdly, An original signal with a white noise is given as follows:

$$s = \sin (x) + \varepsilon$$

where $$\varepsilon \sim N(0,\sigma^{2} )$$.

**Figure 3:**
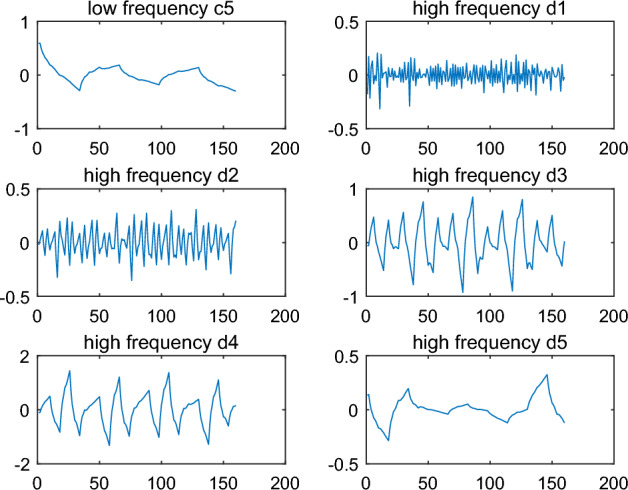
Decomposition of the original signal.

The raw signal $s$ is decomposed onto five level and low frequency coefficient $c_{5}$ and high frequency coefficients $d_{1} ,d_{2} ,d_{3} ,d_{4} ,d_{5}$ can be obtained. The results are shown in Fig. 3.

Fourthly, the array in Step two is taken a linear transform. And the transformed array is added to the high frequency coefficient by choosing the appropriate position. The new high frequency coefficients $d_{{1}}^{*}$ is obtained.

Finally, the coefficient $c_{5}$ and $d_{{1}}^{*} ,d_{2} ,d_{3} ,d_{4} ,d_{5}$ can be reconstructed to generate a new signal $\tilde{s}$ with the array information. The result is shown in Fig. 3.

**Figure 4:**
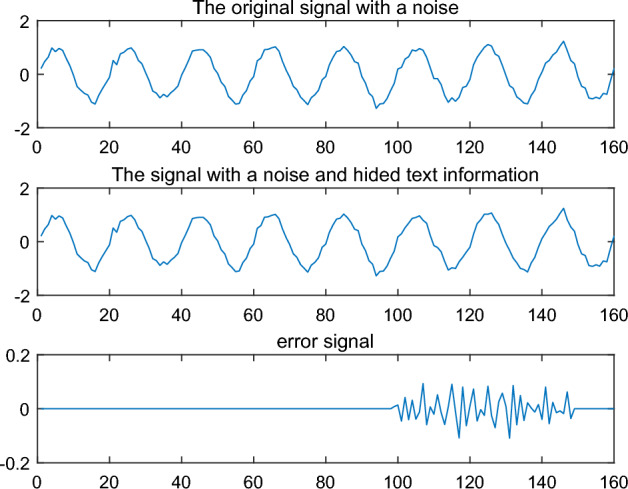
Original signal and the signal with text information.

The above procedure implements that the text information "Text Hiding and Recovery" is hidden in a noise signal. By observing ‘original signal with a noise’and ‘The signal with a noise and hided text information’ directly in Fig. 4, it is not easy to see the difference between them. That means is not easy to observe the hidden text information in the signal directly. By calculating the error signal, a significant error is found from the node 100 to 140 in the signal (see 'error signal' in Fig. 4). So the text information “Text Hiding and Recovery” is hidden between node 100 and node 140.

## Recovery algorithm for text information

In the previous section, the hiding algorithm for text information is introduced by the wavelet transform. In this section, a recovery algorithm is proposed for hidden information.

### Wavelet text recovery algorithm (WTRA)

Step one, the signal $\tilde{s}$ with text information is decomposed onto several levels and low frequency coefficient and high frequency coefficients can be obtained by DWT.

Step two, According to the chosen position, the data containing text information is captured from the designated high frequency.

Finally, according to the obtained data and English letter database, text information can be restored by an transform.

**Figure 5:**
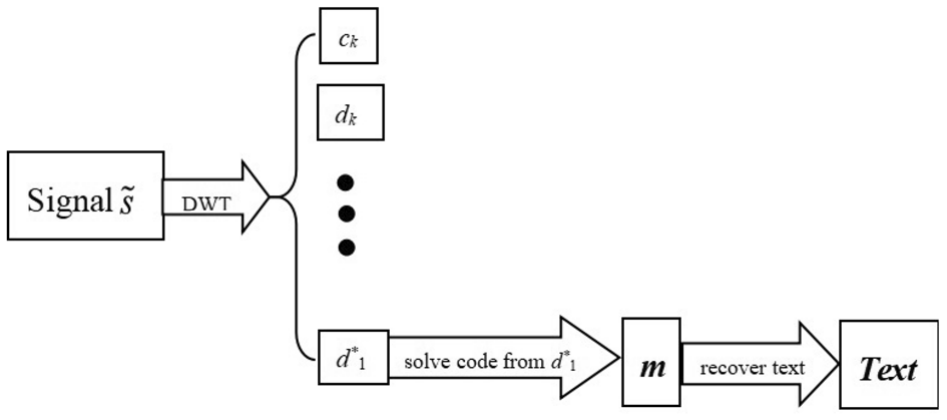
Diagram of recovery algorithm for text information.

The text information hidden in the signal can be restored by the above steps. This approach is called a wavelet text information recovery algorithm. The diagram of this approach is shown in Fig. 5.

Note 3: The obtained data should be taken the inverse linear transform in Step4 of WTHA to recover the initial code.

Next, the following example is given for illustrating wavelet text recovery algorithm.

#### Example 2

In this example, the text ”Text Hiding and Recovery” can be recovered from the synthesized signal $\tilde{s}$ obtained in Example 1.

**Figure 6:**
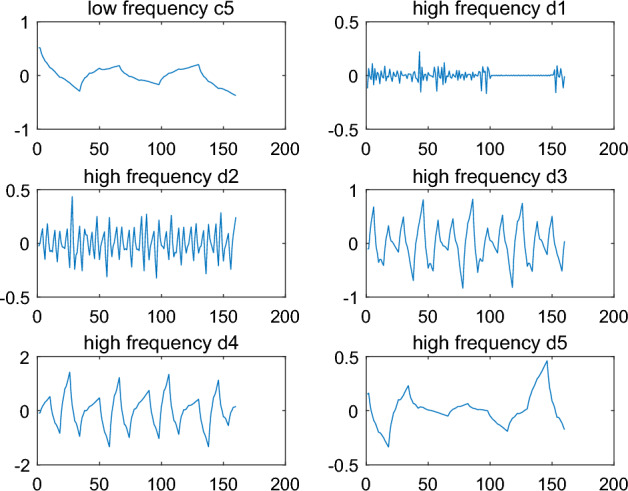
Decomposition of the signal with text information by wavelet ‘db2’.

Firstly, the signal $\tilde{s}$ is decomposed onto five levels by DWT and low frequency coefficient $c_{5}$ and high frequency coefficients $d_{1} ,d_{2} ,d_{3} ,d_{4} ,d_{5}$ can be obtained. The results are shown in Fig. 6. compared to the result in Fig. 3, high frequency coefficients $d_{1}$ of $\tilde{s}$ is different from that of $s$. So text information could be capture by dealing with the data $d_{1}$.

Secondly, according to the information of position in the wavelet text information hiding algorithm, the code array containing text information is captured by dealing with the high frequency $d_{1}$. The array is shown as follows:

‘[20,31,50,46,53,8,35,30,35,40,33,53,27,40, 30,53,18,31,29,41,48,31,44,51]’.

Finally, by the above array, text information can be recovered as

“Text Hiding and Recovery”.

## Characteristics of hiding algorithm and recovery algorithm

**Figure 7:**
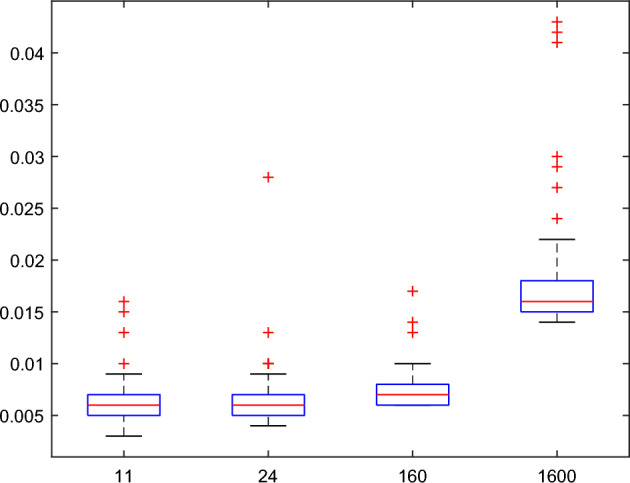
Boxplot of running time distribution for the wavelet text hiding algorithm.

In the wavelet text information hiding algorithm and wavelet text information recovery algorithm, the code of text information is hidden by noise $\varepsilon$. That means the code of text information can not be captured from the noise easily by wavelet denoising method, because the code of text information and noise $\varepsilon$ are mixed together. So noise $\varepsilon$ guarantees the security for the code of the text information. In the wavelet text hiding algorithm and recovery algorithm, the main computation complexity is the complexity of the wavelet transform. If the length *N* of a signal is equal to $k_{0} {2}^{{J_{0} }}$, where *k*_0_ is a positive integer, the result of the DWT can be calculated by $O(N)$ times of multiplication. Thus English text information can be hidden and recovered quickly by the wavelet text hiding algorithm and recovery algorithm respectively. In order to illustrate the running time of the wavelet text hiding algorithm, 1000 English texts with different length are chosen for testing the distribution of running time. All English texts are accurately recovered. A distribution figure of running time is shown in the boxplot of Fig. 7. The *X* axis represents the length of English texts, and the *Y* axis is the running time (in seconds). The mean values of the running time are 0.0065 s, 0.0066 s, 0.0075 s and 0.0173 s corresponding to the length 11, 24, 160 and 1600 respectively. The variances are ${6}.{8561} \times {10}^{ - 6}$,${6}.{8565} \times {10}^{ - 6}$,$2.9595 \times {10}^{ - 6}$ and $2.7465 \times {10}^{ - 5}$ respectively. According to the above discussion, the wavelet text hiding algorithm is quick and stable.

**Figure 8:**
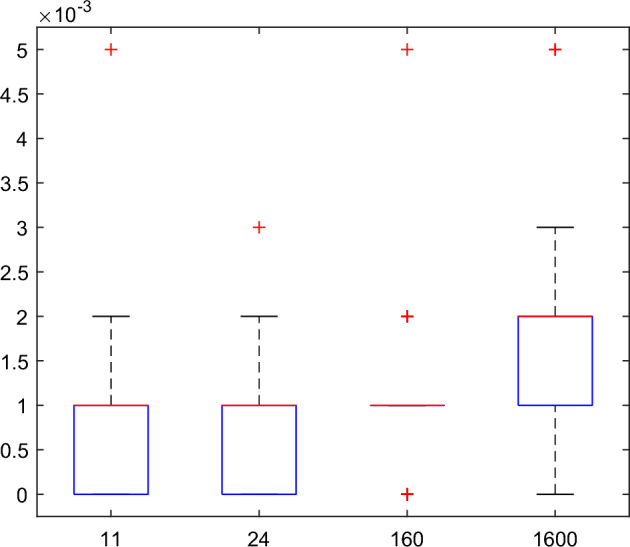
Boxplot of running time distribution for the wavelet text recovery algorithm.

Similarly, the running time distributions for the corresponding wavelet text recovery algorithm are also given in the boxplot of Fig. 8. The mean values of the running time are ${8}{\text{.6000}} \times {10}^{{ - {4}}}$ s, ${8}{\text{.8000}} \times {10}^{{ - {4}}}$ s, ${9}{\text{.7000}} \times {10}^{{ - {4}}}$ s and 0.0016 s corresponding to the length 11, 24, 160 and 1600 respectively. The variances are ${5}{\text{.2566}} \times {10}^{{ - {7}}}$, ${4}{\text{.5010}} \times {10}^{{ - {7}}}$, ${4}{\text{.5364}} \times {10}^{{ - {7}}}$ and ${6}{\text{.2184}} \times {10}^{{ - {7}}}$ respectively. So the wavelet text recovery algorithm is also quick and stable. Compared to the hiding algorithm, the recovery algorithm is quicker.

**Figure 9:**
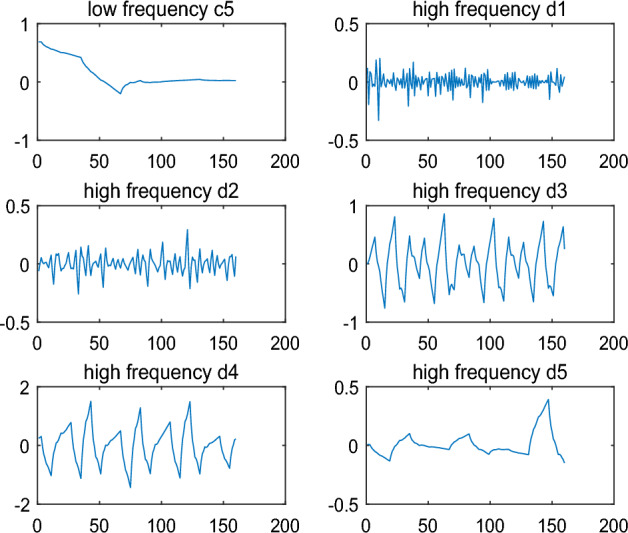
Decomposition of the signal with text information by wavelet ‘db3’.

In these algorithms, the wavelet function can be chosen arbitrarily to hide and recover the text information, and it is also very critical. In text information hiding, any one wavelet function can be applied. However, in text information recovery, the wavelet function must be consistent with that in text information hiding. Otherwise, the text information can not be recovered accurately and quickly. For example, wavelet function ‘db2’ is chosen to hide the text information in Example 1. If wavelet ‘db3’ is chosen to recover the text information, the results of decomposition are shown in Fig. 9. The signal the signal $\tilde{s}$ with text information is also decomposed to five levels by DWT. In Fig. 9, the low frequency coefficient $c_{5}$ and high frequency coefficients $d_{1} ,d_{2} ,d_{3} ,d_{4} ,d_{5}$ are significantly different from that in Fig. 6, especially $d_{1}$. According to the approach in text information recovery, the code is recovered as follows:

‘[6,16,6,4,7,4,20,8,13,2,4,5,4,7,1,6,5,2,6,4,10,2,5].’

The text information corresponding to the code is

‘FPFDGDTHMBDEDGAFEBFDJBE’.

That means the recovery of text information is failed. This is because the wavelet function in the recovery algorithm is inconsistent with that in the hiding algorithm.

In order to recover the text information from the signal $\tilde{s}$, it need to be known in which high-frequency coefficient, the coding is hidden. If this information is not known, more time is needed to recover the code. Moreover, The level number of wavelet decomposition is also needed to be known, because the decomposed level number of signal $\tilde{s}$ is $\left[ {\log_{2} N} \right]$, where $N$ is the length of the signal $\tilde{s}$ and $\left[ \cdot \right]$ is an integral function. The last important information is the position in which the transformed code of text information is added to the high frequency coefficient. The position in the embedded signal can be either continuous or intermittent. If it is continuous, the different embedding has $N - l$ results, where $l$ is the length of the code of text information. If it is intermittent, the different embedding has $P_{N}^{l}$ results, where $P_{N}^{l}$ is a permutation number, $P_{N}^{l} = \frac{N!}{{l!}} = N(N - 1)(N - 2) \ldots (N - l + 1)$.

**Figure 10:**
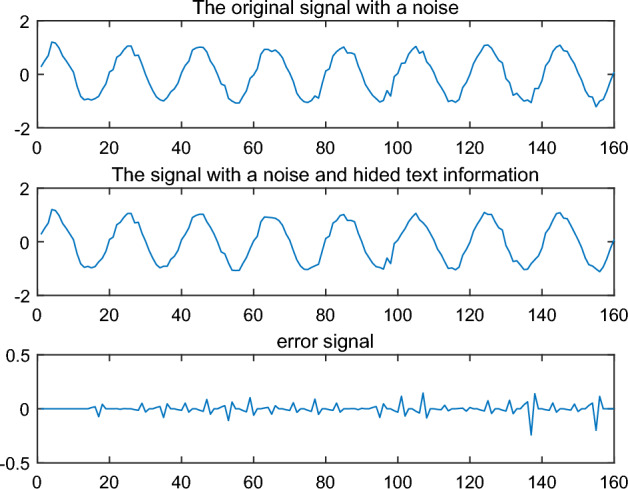
Original signal and the signal with text information by the intermittent embedding.

**Figure 11:**
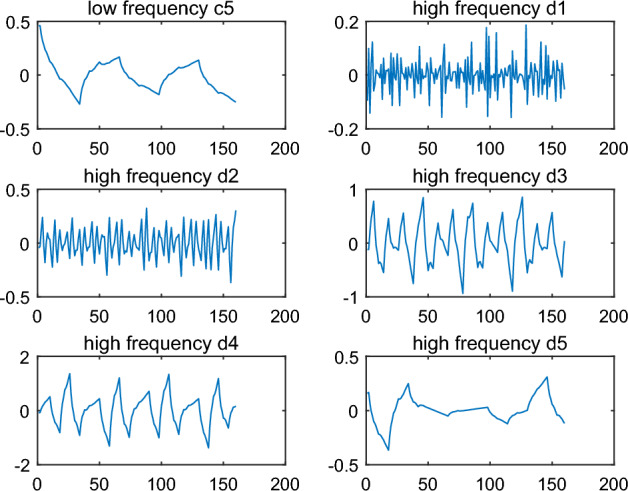
Decomposition of the signal with text information by the intermittent embedding.

An example is given for illustrating the intermittent embedding. The result $\tilde{s}$, in which the text code is embedded the signal $s$ in Example 1, is shown in Fig. 10. Compared to the Fig. 4, it is easy to see that the text code is mixed in error signal and it is difficult to identify the embedded position. The low frequency coefficient $c_{5}$ and high frequency coefficients $d_{1} ,d_{2} ,d_{3} ,d_{4} ,d_{5}$ are shown in Fig. 9 by wavelet decomposition. Compared to the $d_{1}$ in Fig. 3 and 6, there are too many differences in Fig. 11 to determine exactly which positions are different in $d_{1}$. Thus, in the case of the intermittent embedding, the code is hard to be recovered from high frequency coefficient $d_{1}$ without the embedded position. In a word, these critical points can constitute a private key.

According to the above discussion, wavelet text hiding algorithm and wavelet text recovery algorithm can be recognized as a public key cryptography for text information.

Public key: Signal $\tilde{s}$, Length *l* of the text.

Private key: a private key consists of wavelet function (db2), number of decomposition level (5) and embedding position (d1, continuous embedding position in d1).

Plaintext: Text Hiding and Recovery.

According to the above discussion, this method is consistent with the public key mechanism and has the following characteristics. Firstly, it has two kinds of keys, the public key is public, and the private key is secret. Secondly, deriving a private key from the public key is not computationally feasible. Thirdly, the information encrypted with the public key must be decrypted by a relative private key. Finally, the information encrypted with a private key must be decrypted by using the corresponding public key. According to these critical points, a personalized private key can be also designed by designers or users. These critical points can ensure the security of the text information. Since there are many variables for invaders to be considered, it is very difficult to decipher the text. Not all keys need to be transmitted, in the algorithm, what need to be transmitted is only the synthetic signal and the length *l*. This approach is similar to a ‘lock’ with only one ‘key’. Public keys turn off the text message into a ‘lock’. All critical points form a useful ‘key’, where each critical point is equivalent to a bump on the ‘key’. Only if every critical point is correct, the text message can be obtained. Especially, if the wavelet filters is designed by some new algorithm, the text message is almost impossible to be deciphered, even knowing the hidden algorithm.

**Figure 12:**
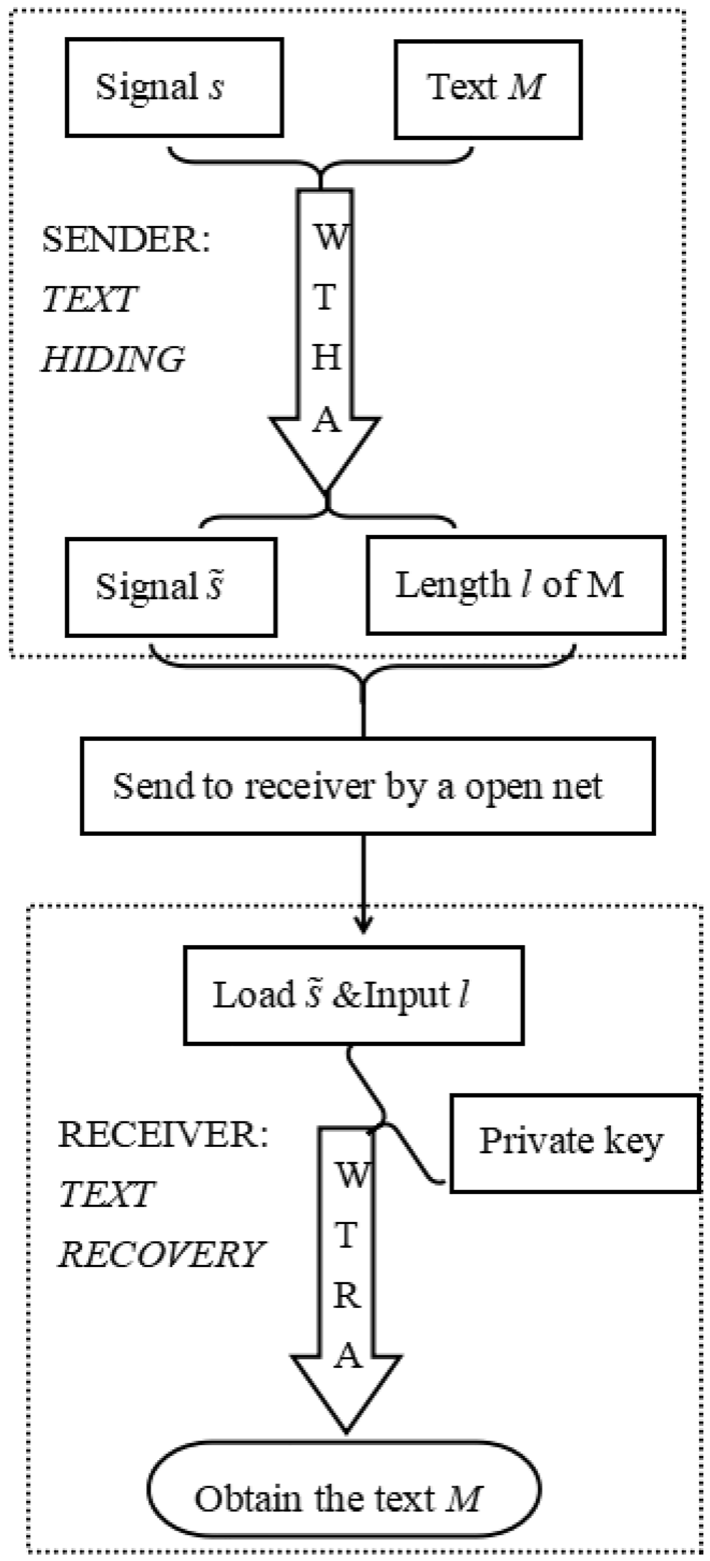
System architecture figure.

Thus, a complete set of text encryption transmission approach can be proposed by wavelet text hiding algorithm (WTHA) and wavelet text recovery algorithm (WTRA). A system architecture figure for the proposed approach is shown as the following Fig. 12. This system includes sender (*TEXT HIDING*) and receiver (*TEXT RECOVERY*). For a sender, a synthetized signal $\tilde{s}$ can be generated form a signal *s* and text *M* by the WTHA and the length *l* of *M* can obtained. Through the public network, the synthetized signal $\tilde{s}$ and the length *l* can be transmitted to a receiver by sender. For the receiver, the WTRA can be run by the private key to recover the text *M* from the obtained synthetized signal $\tilde{s}$ and the length *l*.

Note 4: In the public network transmission, if some non-critical data of $\tilde{s}$ is tampered by others, text *M* can still be recovered. If some critical data of *M* in $\tilde{s}$ is tampered by others, part of text *M* can be recovered. But if all critical data of *M* in $\tilde{s}$ is tampered, the text *M* can not be recovered. It implicates that this system has some ability to resist data tampering.

## Conclusion and discussion

Based on the wavelet digital watermarking method, an approach is given for hiding some text information in a signal with a white noise. An example and some figures are shown for illustrating how to hide text information in a signal with white noise $\varepsilon$. the noise $\varepsilon$ guarantees the security for the code of the text information. Moreover, a method is proposed to recover the text information from the synthetic signal. In order to recover the text information correctly, critical information must be known privately, including wavelet function, number of decomposition level, embedding position. The usual digital watermark is used to protect digital product copyright, integrity, replication or tracking, such as digit images, video, audio or electronic documents. The idea of the algorithm, which is hiding text information via wavelet digital watermarking method, to protect the watermark by a digital signals with noise. And the watermark is the important text message, which is need to be transmitted to others. In addition, this English text hiding approach and recovery method cannot be done by manual calculation, and can only be done by using a computer, unlike the Morse code. This is both a merit and a problem. How to hide and recover the English text by manual calculation in extreme cases. This will be a very challenging research question in the future. Of course, there are many encoding methods. Alphabetical is one of simplest method. So the code of text information or the embedded location can be encrypted to improve security. Moreover, with the advent and development of GPT, whether this approach can maintain its security. In other words, how to improve the algorithm makes the GPT undecipher in limited time. That means even if the GPT knows that the algorithm, it cannot be deciphered. Moreover, Based on the wavelet space, it is recently discovered that many different types of data might been hidden at once. This is more interesting. The further exploration in theory is still under study. These new problems and challenges will be continued in our follow-up study.

## Data Availability

All data generated or analyzed during this study are included in this published article and the uploaded file ‘data.xls’.
